# Visual, Vestibular, and Somatosensory Function in Female Rugby League Athletes

**DOI:** 10.3390/sports14070265

**Published:** 2026-06-26

**Authors:** Riley Brassington, Jocelyn Mara, Nick Ball, Gordon Waddington, Julie Cooke

**Affiliations:** Research Institute of Sport and Exercise, Department of Sport and Exercise Science, Faculty of Health, University of Canberra, Bruce, ACT 2617, Australia

**Keywords:** proprioception, visual–vestibular function, pupillary light reflex, female rugby league

## Abstract

Female rugby league performance is influenced by multiple interacting sensory and physiological systems; however, the extent to which these factors vary across playing levels and positional groups remains unclear. This study explored differences in visual, vestibular, somatosensory, and autonomic performance according to playing level and position in female rugby league athletes. Elite and sub-elite athletes completed lower-limb proprioception testing using the Active Movement Extent Discrimination Assessment protocol alongside visual-vestibular and autonomic assessments obtained via a virtual reality eye-tracking system. Bayesian hierarchical models examined the effects of playing level, positional group (adjustables, backs, forwards), and their interaction. Few consistent differences were observed between elite and sub-elite athletes across the measures assessed. Posterior estimates suggest selected level-by-position effects for ankle proprioceptive acuity (PD = 0.94), vestibulo-oculomotor time on target (PD = 0.95), and autonomic dilation velocity (PD = 0.98); however, these findings were not consistent across positional groups or outcome measures, and within-group variability was evident. Overall, sensory and autonomic performance did not consistently differentiate elite and sub-elite athletes, suggesting limited utility as cross-sectional markers of playing level but potential value as longitudinal monitoring tools alongside workload, recovery, and performance data.

## 1. Introduction

Lower limb proprioception, the ability to sense joint position and movement, forms the foundation of balance control and is essential for skilled athletic performance. Research consistently shows that superior proprioceptive acuity at the lower limb is associated with higher levels of competition, better agility, balance, and technical execution [1,2]. Higher-ranked athletes demonstrate superior proprioceptive performance compared with lower-ranked athletes and non-athletic populations, while proprioceptive acuity has also been identified as a predictor of progression to the highest levels of competition, including international and Olympic representation [2]. In contrast, deficits in proprioception elevate the risk of lower limb injury, ankle sprains, and chronic instability, which not only impair immediate performance but also predispose athletes to recurrent injuries that may shorten athletic careers [3,4,5].

Beyond its role in injury prevention, proprioceptive acuity is fundamental to whole-limb coordination and the execution of complex athletic movements [6]. Efficient proprioceptive feedback supports precise foot placement, joint alignment, and the integration of multiple joints during high-speed, multi-planar actions such as sprinting, tackling, and rapid changes in direction [6,7,8]. When proprioceptive processing is well developed, movement control becomes more automatic, reducing the cognitive load required for motor regulation and allowing greater attentional resources to be allocated to higher-order tasks such as tactical awareness and decision-making [9]. Consistent with this, proprioceptive training interventions have been shown to enhance postural stability, agility, and explosive strength while reducing the incidence of ankle injuries across athletic populations [4,6,8,10,11,12]. Collectively, this evidence highlights proprioceptive acuity as a key contributor not only to injury risk reduction but also to movement efficiency and long-term athletic development in team collision sports.

Recent evidence from multisensory athlete assessment further suggests that integrated visual, vestibular, somatosensory, and autonomic measures may provide complementary insight into sensorimotor characteristics associated with performance level when interpreted alongside broader coaching and performance information [13]. Together, these systems underpin coordinated movement control, allowing athletes to perform with precision in high-speed, rapidly changing sporting environments.

Rugby league presents a particularly demanding context for multisensory integration. Players perform frequent accelerations, decelerations, directional changes, and collisions while fatigued, requiring constant recalibration of visual, vestibular, and proprioceptive input. Positional demands may further influence sensory reliance; forwards engage predominantly in close-contact and wrestling actions, potentially placing greater emphasis on proprioceptive and vestibular inputs to maintain stability and technique, whereas backs perform high-speed evasive manoeuvres in open play, relying more heavily on visual processing, gaze stabilisation, and precise limb coordination to track opponents and the ball. Optimal integration of these sensory systems may enhance movement efficiency and performance consistency, while impairments may contribute to reduced postural control and increased susceptibility to injury [14].

Based on the importance of proprioception in performance and injury risk reduction, reliable assessment of sensory performance is vital. The Active Movement Extent Discrimination Assessment (AMEDA) protocol provides an ecologically valid weightbearing measure of lower-limb somatosensory acuity at the ankle [15]. In addition to demonstrating acceptable reliability across healthy and clinical populations, the AMEDA protocol has shown evidence of discriminant validity through its ability to distinguish between individuals with chronic ankle instability and healthy controls, younger and older adults, and neurological populations [16,17]. Furthermore, AMEDA performance has been associated with balance performance, injury status, and competitive sporting level, supporting its utility as a functional measure of ankle proprioceptive acuity [1]. Previous research has also demonstrated superior proprioceptive performance in higher-performing and elite athletes compared with lower-level athletes, suggesting that the protocol may be sensitive to expertise-related differences in somatosensory function [2].

Recent technical advances have expanded this approach through the integration of visual and vestibular assessments using a programmable headset, enabling the evaluation of smooth pursuit, saccadic eye movements, and pupillary light reflex (PLR). This combined assessment framework has demonstrated acceptable reliability and preliminary validity in a general population [18]. While the use of virtual reality and eye-tracking technologies for visual-vestibular assessment is supported by evidence demonstrating construct validity and diagnostic utility in related systems, evidence regarding the ability of the Prism Neuro assessment framework to distinguish between athletes of differing expertise or playing levels remains limited [19]. Consequently, the potential utility of these measures for differentiating playing levels in applied sporting environments is not yet well understood. Clarifying this issue may provide preliminary insight into whether visual-vestibular and autonomic measures can distinguish playing level or contribute to athlete monitoring in applied sport settings.

Despite evidence linking proprioceptive acuity and multisensory integration to athletic performance and injury risk, little is known about how these sensory systems vary across playing levels and positional groups in rugby league athletes. To date, no study has concurrently examined proprioceptive, visual, vestibular, and autonomic function across playing levels and positions within rugby league. Examining these measures in female rugby league athletes may inform targeted monitoring, training, and injury-prevention strategies, while clarifying their potential role within applied athlete monitoring frameworks.

Therefore, the aim of this study was to investigate differences in visual, vestibular, somatosensory, and autonomic performance across playing levels and positional groups in female rugby league athletes. Given the established relationship between proprioceptive acuity and competitive playing level, but limited evidence regarding visual-vestibular and autonomic function in female collision-sport athletes, the latter domains were explored in a more exploratory capacity. Based on previous evidence linking proprioceptive acuity with competitive level, it was hypothesised that elite athletes would demonstrate superior somatosensory performance compared with sub-elite athletes. Given the limited evidence regarding visual-vestibular and autonomic function in female collision-sport athletes, these outcomes were examined in an exploratory capacity. No directional hypothesis was proposed for positional group effects.

## 2. Materials and Methods

### 2.1. Participants

Participants were recruited from one elite National Rugby League Women’s (NRLW) team and one sub-elite Tarsha Gale Cup team, representing the same professional rugby league club. Owing to the relatively small number of contracted athletes within the NRLW competition, the available participant pool was limited; consequently, it was necessary to follow a single team across two consecutive seasons to obtain a sufficient sample for analysis. The Tarsha Gale Cup is the premier under-19 female rugby league competition within the NSW/ACT region. All participants were injury-free at the time of testing. Athletes with acute musculoskeletal injuries or unresolved concussion symptoms were excluded by the medical staff. Vision correction (e.g., glasses or contact lenses) was permitted, provided it was worn consistently during testing. All participants provided written informed consent prior to testing.

Anthropometric measures (age, height, and body mass) were recorded for both groups as part of routine preseason baseline testing, conducted by trained staff using standardised procedures (Table 1).

### 2.2. Study Design

This study employed a cross-sectional observational design involving multiple cohorts. Ethics approval was obtained from the university’s Human Research Ethics Committee prior to data collection (HREC–12219). Data were collected during the 2023 and 2024 preseason periods from athletes representing one elite NRLW team and one sub-elite Tarsha Gale Cup team. Testing occurred in week 1 and week 8 of each team’s preseason schedule, approximately eight weeks apart. During this period, both squads followed a broadly similar training structure comprising three scheduled team sessions per week that included field-based training, with resistance training incorporated into two sessions. A total of 80 unique athletes (47 sub-elite and 33 elite) contributed 174 observations across the study period. Although squad composition changed across testing occasions, a subset of athletes participated in multiple testing sessions. Accordingly, athlete ID and testing occasion were included as random effects within the Bayesian hierarchical models to account for repeated observations where present.

### 2.3. Data Collection and Procedures

All assessments were conducted in a controlled indoor environment designed to minimise auditory and visual distractions and ensure consistency across sessions. To maintain standardised administration and data integrity, all testing was performed by the same experienced assessor. Testing was conducted as part of routine preseason athlete monitoring. No formal assessment of fatigue, sleep quality, or recovery status was undertaken prior to testing. Menstrual cycle information was unavailable because no centralised monitoring system was utilised by the participating teams during the study period. The AMEDA protocol and Visual-Vestibular System (VVS); Prism Neuro^®^ Pty Ltd., Canberra, Australia. procedures used in this study have previously demonstrated strong test–retest reliability, and consistent administration was maintained across both testing sessions [18]. Each participant completed two consecutive five-minute assessments: a visual-vestibular system (VVS) evaluation using a virtual reality video headset, and a somatosensory assessment.

### 2.4. Visual-Vestibular System (VVS) Assessment

The VVS protocol utilised a Prism Neuro^®^ Pty Ltd. headset equipped with dual OLED displays (2560 × 1440 resolution), a 70 FPS refresh rate, a 100° field of view, and a 120 Hz infrared eye-tracking system with a reported median accuracy of 1.15° across the screen. Participants underwent a standardised calibration process to map gaze positions across the visual field.

Following calibration, three eye-tracking trials were administered in a fixed sequence to control for light adaptation effects:

1. Smooth pursuit: Participants tracked a small red dot moving in a circular path.
2. Pupillary response: Participants focused on a stationary red ‘x’ while lights flashed, allowing measurement of pupil constriction and dilation velocities.
3. Saccadic movement: Participants tracked a rapidly moving red ‘x’ across the screen.

Standardised instructions were provided to ensure consistency. Data were processed using proprietary blink filtering and low-pass signal smoothing algorithms.

### 2.5. Somatosensory Assessment

Ankle proprioceptive acuity was assessed using a Prism Neuro AMEDA system, a validated tool for evaluating somatosensory sensitivity during weight-bearing tasks [18]. Participants stood barefoot on the device, placing one foot on a moveable platform and the other on a fixed platform. The moveable platform rotated into five distinct inversion angles (approximately 11° to 15° in 1° increments), labelled 1 through 5. A familiarisation phase was completed on the left limb, during which participants performed three trials at each inversion depth (15 movements total). Movements were self-paced, with participants actively inverting the ankle until the platform reached a mechanical stop, then returning to neutral. Following familiarisation, participants completed 50 randomised trials per limb (10 trials per inversion depth). After each movement, they retrospectively identified the perceived position number (1–5) without feedback. Participants maintained a forward gaze and relaxed posture to minimise extraneous sensory input. Responses were manually recorded using an Android tablet (Samsung Electronics Co., Ltd., Suwon, Republic of Korea) and transferred to Microsoft Excel. Somatosensory acuity was quantified using the area under the curve (AUC) of a Receiver Operating Characteristic (ROC) analysis, with scores ranging from 0.5 (chance level) to 1.0 (perfect discrimination).

### 2.6. Statistical Analysis

Statistical analyses were conducted using RStudio (Version 2024.09.0 + 375). Descriptive statistics, including mean and standard deviation (SD), were calculated for all continuous variables. To examine the effects of playing level (elite vs. sub-elite) and positional group (adjustables, backs, forwards) on sensory performance outcomes, Bayesian hierarchical models were fitted using the ‘brms’ package. Each model specified a Gaussian likelihood and included fixed effects for level, position, and their interaction, along with random intercepts for athlete ID and testing occasion to account for repeated measures and nested data structure. Bayesian hierarchical modelling was selected because it accommodated the repeated observations, unbalanced positional groups, and hierarchical structure of the dataset while enabling estimation of uncertainty around parameter estimates. Posterior distributions were summarised using median estimates and 95% highest density intervals (HDIs). Directional probabilities (PD) were calculated to assess the likelihood of effects being positive or negative [20]. Practical significance was evaluated using Region of Practical Equivalence (ROPE) analysis, with thresholds defined as ±0.1 standard deviations of the respective response variable. ROPE percentages were used to interpret the proportion of the posterior distribution falling within the equivalence bounds. Estimated marginal means (EMMs) were computed using the ‘emmeans’ package, and pairwise contrasts were extracted using posterior draws [21]. Model convergence was assessed using R-hat statistics (<1.01) and effective sample sizes, with all models demonstrating satisfactory convergence. Uniform priors were used for all fixed effects.

## 3. Results

### 3.1. Somatosensory Variables

There was evidence of a level × position interaction effect for AMEDA Left, indicating that the effect of level depended on position (β [Level × Backs vs. Adjustables] = 5.8, 95% HDI = [−1.6, 13.3], PD = 0.94). Specifically, elite adjustables had higher AMEDA Left scores than sub-elite adjustables (β = 3.7, 95% HDI = [−2.0, 9.6], PD = 0.89), but there was limited evidence of a difference between levels for backs or forwards (Table 2).

On the other hand, there was no interaction effect (β [Level × Backs vs. Adjustables] = 2.7, 95% HDI = [−3.2, 9.0], PD = 0.80; β [Level × Forwards vs. Adjustables] = 1.1, 95% HDI = [−4.8, 6.6], PD = 0.65) or main effect for level for AMEDA Right (B = −1.3, 95% HDI = [−5.9, 3.5], PD = 0.70).

### 3.2. Vestibulo-Oculomotor Variables

There was no clear level × position interaction effect for time to target (β [Level × Backs vs. Adjustables] = 0.02, 95% HDI = [−0.05, 0.09], PD = 0.69; β [Level × Forwards vs. Adjustables] = 0.01, 95% HDI = [−0.05, 0.07], PD = 0.62), indicating that the effect of competition level did not differ meaningfully across positional groups (Table 2). There was also no main effect of level when averaged across positions (β = 0.01, 95% HDI = [−0.05, 0.06], PD = 0.60).

There was a level × position interaction effect for time on target, indicating that the effect of competition level depended on positional group (β [Level × Backs vs. Adjustables] = 0.11, 95% HDI = [−0.02, 0.22], PD = 0.95). Elite adjustables achieved higher time on target scores than sub-elite adjustables (β = 0.08, 95% HDI = [−0.01, 0.17], PD = 0.89), whereas sub-elite backs achieved higher scores than elite backs (β = −0.11, 95% HDI = [−0.22, 0.02], PD = 0.95) (Table 2).

There was no clear level × position interaction effect for circular tracking error (β [Level × Backs vs. Adjustables] = −0.05, 95% HDI = [−8.04, 7.49], PD = 0.50; β [Level × Forwards vs. Adjustables] = 4.07, 95% HDI = [−3.25, 10.60], PD = 0.88), indicating similar effects of competition level across positional groups (Table 2). Likewise, there was no main effect of level for circular tracking error (β = 1.02, 95% HDI = [−6.9, 4.9], PD = 0.63).

### 3.3. Autonomic Variables

There was no clear level × position interaction effect for response speed (β [Level × Backs vs. Adjustables] = 3.28, 95% HDI = [−33.96, 21.69], PD = 0.60; β [Level × Forwards vs. Adjustables] = 3.41, 95% HDI = [−28.88, 21.13], PD = 0.61), indicating that competition level had a similar effect across positional groups (Table 2). There was also no main effect of level when averaged across positions (β = 8.85, 95% HDI = [−30.05, 12.51], PD = 0.80).

There was a level × position interaction effect for dilation velocity, indicating that the effect of competition level differed by position (β [Level × Backs vs. Adjustables] = 0.18, 95% HDI = [0.03, 0.36], PD = 0.98). Elite backs demonstrated higher dilation velocity scores than sub-elite backs (β = 0.07, 95% HDI = [−0.07, 0.18], PD = 0.84), while no clear differences between levels were observed for adjustables or forwards (Table 2).

There was a level × position interaction effect for peak constriction velocity, indicating that the effect of competition level depended on positional group (β [Level × Backs vs. Adjustables] = 0.60, 95% HDI = [−0.24, 1.38], PD = 0.92). Elite adjustables demonstrated higher peak constriction velocity scores than sub-elite adjustables (β = 0.28, 95% HDI = [−0.33, 0.97], PD = 0.82). In contrast, sub-elite backs achieved higher peak constriction velocity scores than elite backs (β = 0.40, 95% HDI = [−0.16, 1.03], PD = 0.89), with no clear differences observed between levels for forwards (Table 2).

## 4. Discussion

This study explored differences in visual, vestibular, somatosensory, and autonomic performance according to playing level and positional grouping in female rugby league athletes. Despite evidence supporting the importance of sensory and autonomic function in athletic performance, few consistent differences were observed between elite and sub-elite athletes across the measures assessed. Although some outcomes favoured elite athletes, these effects were generally inconsistent across positional groups and sensory domains. Collectively, these findings suggest that playing level alone may not be a primary determinant of sensory or autonomic function within trained female rugby league athletes.

Importantly, the present findings indicate that sensory and autonomic performance did not consistently differ between elite and sub-elite athletes across positional groups. Instead, differences were task- and position-specific, and variability existed within both elite and sub-elite cohorts. These findings suggest that visual, vestibular, somatosensory, and autonomic function may have limited utility as standalone indicators of competition level. The observed variability may also reflect unmeasured contextual factors, including positional demands, training exposure, recovery status, and individual physiological differences.

**Figure 1:**
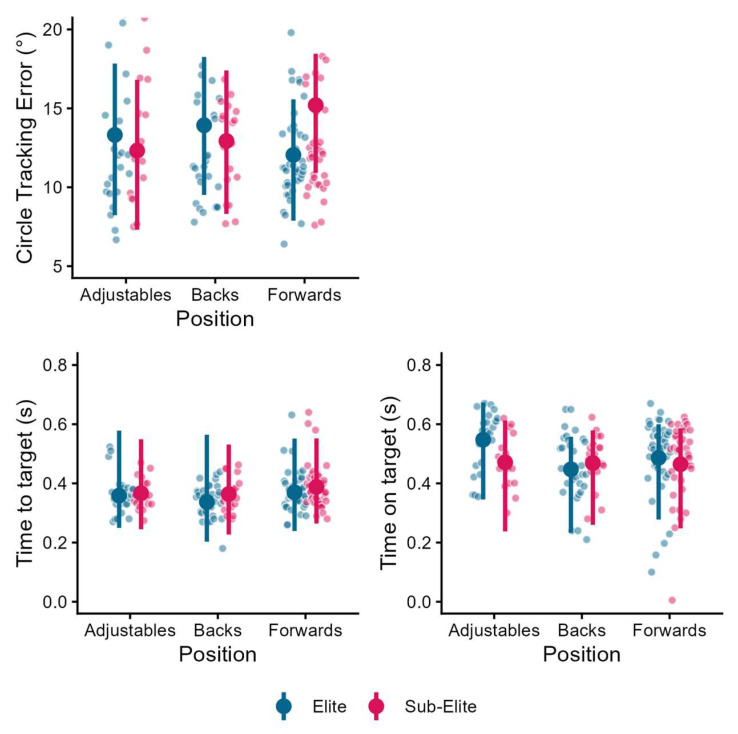
Vestibulo-oculomotor performance of elite and sub-elite female rugby league athletes assessed via Prism Neuro eye-tracking. Circle tracking error (°); time to target (s); time on target (s), presented by positional group (adjustables, backs, forwards). Faded points represent individual observations. Solid points represent model-estimated marginal means, with error bars indicating 95% highest density intervals (HDIs).

Response speed, defined as the latency from light stimulus onset to pupil constriction initiation, serves as an indicator of autonomic integration and neural conduction efficiency, with shorter latencies reflecting more rapid processing [13]. Although elite forwards demonstrated trends toward faster response speeds and higher pupil dilation velocities compared with sub-elite forwards, the magnitude and certainty of these differences were modest (Figure 1). While these observations should be interpreted cautiously, they may indicate that autonomic measures are sensitive to contextual influences such as training exposure, fatigue, and recovery status. Future longitudinal studies may help clarify whether these measures represent stable characteristics of playing level or transient responses to physiological state. Existing literature indicates that higher-level athletes may demonstrate enhanced autonomic and neural responsiveness during cognitively demanding tasks [22,23,24]; however, within relatively homogeneous athletic cohorts, such differences may be attenuated or influenced by contextual factors such as training phase and cumulative load.

**Figure 2:**
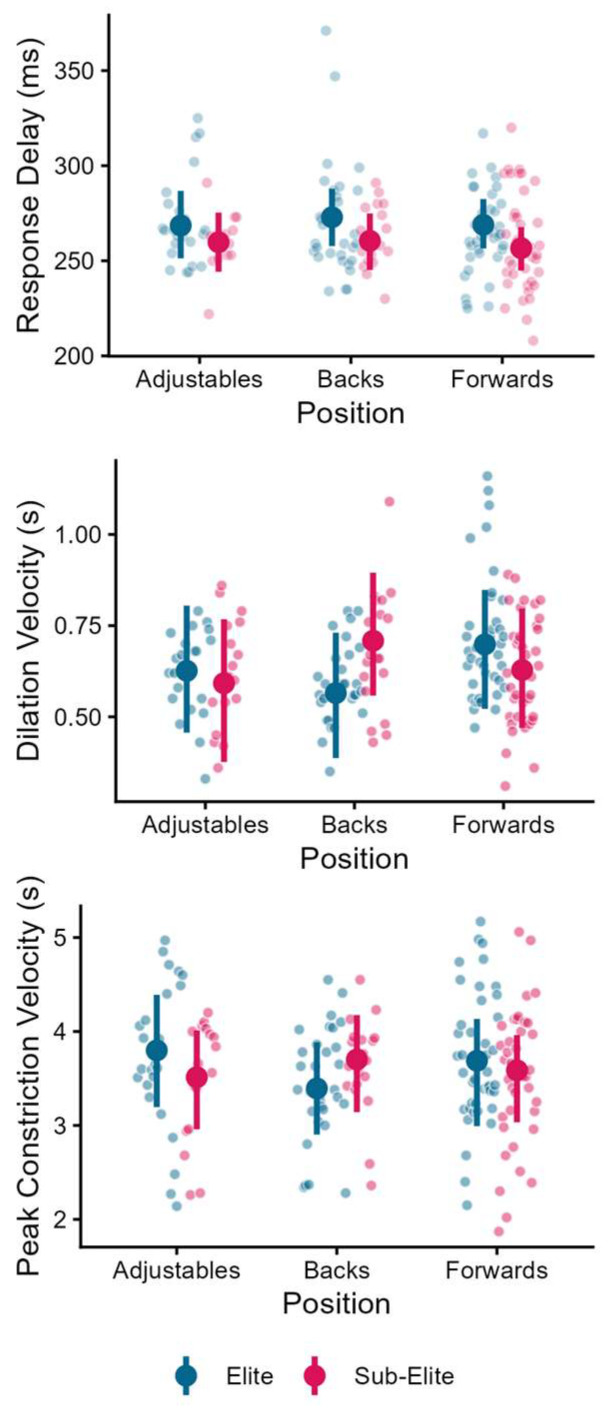
Autonomic performance of elite and sub-elite female rugby league athletes assessed via Prism Neuro eye-tracking. Response delay (ms); dilation velocity (s); peak constriction velocity (s), presented by positional group (adjustables, backs, forwards). Faded points represent individual observations. Solid points represent model-estimated marginal means, with error bars indicating 95% highest density intervals (HDIs).

In contrast, sub-elite backs demonstrated higher parasympathetic and sympathetic PLR metrics compared with elite backs (Figure 2). Although unexpected, both cohorts were assessed late in the pre-season, a period characterised by elevated training loads and accumulated fatigue. Elite athletes are typically exposed to higher absolute training loads during this phase, which may influence autonomic responses at the time of testing. The PLR, governed by parasympathetic (constriction) and sympathetic (dilation) pathways, is recognised as an indicator of neurological and physiological status [25]. Accordingly, these findings may reflect differences in physiological state rather than inherent competition-level characteristics. As these findings were not consistently observed across all positional groups or autonomic variables, further investigation is required before attributing these differences to competition level alone.

**Figure 3:**
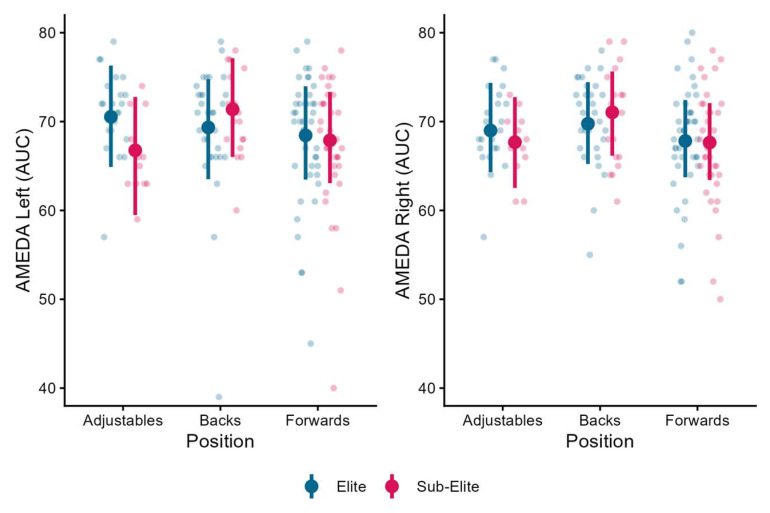
Ankle proprioceptive acuity of elite and sub-elite female rugby league athletes assessed using the AMEDA protocol. AMEDA Left (AUC); AMEDA Right (AUC), presented by positional group (adjustables, backs, forwards). Faded points represent individual observations. Solid points represent model-estimated marginal means, with error bars indicating 95% highest density intervals (HDIs).

Ankle somatosensory acuity, assessed using the AMEDA protocol, showed no consistent differences between elite and sub-elite players and no clear separation across positional groups (Figure 3). This contrasts with previous studies reporting superior proprioceptive acuity in elite compared with lower-level or non-athletic populations [2]. Within trained athletic cohorts, however, shared exposure to sport-specific training and neuromuscular demands may result in broadly similar proprioceptive capability, thereby reducing the ability of the AMEDA protocol to discriminate between playing levels.

The findings should be interpreted within the context of preseason testing, during which elevated training loads and accumulated fatigue may have influenced sensory and autonomic function. Although both squads followed a similar weekly training structure consisting of three team sessions that included field-based training, with resistance training incorporated into two sessions per week, differences in training intensity, cumulative load, and individual responses to training may have contributed to variability in the observed outcomes.

Recovery status was not formally assessed prior to testing, and factors such as sleep quality, psychological stress, and residual fatigue may have influenced performance across sensory and autonomic domains. Menstrual cycle phase and reproductive hormone status were not monitored and may represent a source of unexplained variability, given their potential influence on neuromuscular performance, autonomic regulation, and sensory processing. Additionally, the study was conducted within a single rugby league club, involving elite athletes from an NRLW squad and sub-elite athletes from an under-19 squad during two preseason periods, which may increase the influence of individual variability and limit the generalisability of the findings to other clubs, competitions, or stages of the season. Nevertheless, the consistency of the testing environment, assessment procedures, and training structure across cohorts strengthens confidence that the observed findings reflect meaningful variation within this athlete population.

Practically, the limited and inconsistent level-based differences observed in this study suggest that sensory and autonomic measures may have limited utility as standalone tools for distinguishing playing level in female rugby league athletes. While selected differences were observed across somatosensory, vestibulo-oculomotor, and autonomic domains, these findings were not consistently evident across positional groups or outcome measures. Consequently, sensory and autonomic assessments should be interpreted alongside broader performance, workload, and recovery information rather than as isolated indicators of playing level. Future longitudinal research is required to determine whether these measures are more useful for monitoring changes in athlete status across a season than for differentiating competition level at a single timepoint.

## 5. Conclusions

This study provides an initial exploration of visual, vestibular, somatosensory, and autonomic function across playing levels and positional groups in female rugby league athletes in a single club setting across two seasons. Overall, these measures did not consistently differentiate elite and sub-elite athletes, suggesting that competition level alone may not be a strong determinant of sensory or autonomic performance within trained female rugby league cohorts. Although selected level-by-position differences were observed, these effects were not consistent across sensory domains, outcome measures, or positional groups. Collectively, the findings suggest that visual, vestibular, somatosensory, and autonomic assessments may have limited utility as standalone cross-sectional markers of playing level. However, they may provide useful complementary information when interpreted alongside workload, recovery, and performance data. Longitudinal research is needed to clarify whether these measures are sensitive to changes in athlete status, training exposure, and performance development across a season.

## Figures and Tables

**Table 1:** Participant Characteristics.

Variable	Sub-Elite (n = 47)	Elite (n = 33)
Age (mean ± SD)	17.8 ± 0.6	24.0 ± 4.1
Height (mean ± SD)	167.5 ± 6.5 *	167.8 ± 6.8
Weight (mean ± SD)	71.0 ± 11.7 *	77.5 ± 11.8
Adjustables [*n* athletes (*n* datapoints)]	9 (17)	7 (24)
Backs [*n* athletes (*n* datapoints)]	11 (19)	9 (31)
Forwards [*n* athletes (*n* datapoints)]	27 (40)	17 (43)

* Measured from *n* = 34 athletes due to incomplete data.

**Table 2:** Contrast for different somatosensory, visual-vestibular, and autonomic variables.

Variable	Contrast	Estimate	Lower 95% HDI	Upper 95% HDI	PD	ROPE
**AMEDA Left** **(AUC)**	Elite **Adjustables**–Sub-Elite Adjustables	3.70	−2.21	9.36	0.89	0.08
	Elite Backs–Sub-Elite Backs	−2.02	−6.90	2.19	0.82	0.16
	Elite Forwards–Sub-Elite Forwards	0.52	−2.77	4.15	0.61	0.29
**AMEDA Right** **(AUC)**	Elite Adjustables–Sub-Elite Adjustables	1.32	−3.46	5.96	0.70	0.16
	Elite Backs–Sub-Elite Backs	−1.31	−5.04	2.73	0.76	0.19
	Elite Forwards–Sub-Elite Forwards	0.20	−2.68	3.22	0.55	0.31
**Circle Tracking Error (Degrees °)**	Elite Adjustables–Sub-Elite Adjustables	1.02	−4.80	6.98	0.63	0.19
	Elite Backs–Sub-Elite Backs	1.11	−3.63	6.19	0.66	0.20
	Elite Forwards–Sub-Elite Forwards	−3.15	−6.70	0.47	0.95	0.07
**Time to Target** **(Seconds)**	Elite Adjustables–Sub-Elite Adjustables	−0.01	−0.06	0.05	0.60	0.18
	Elite Backs–Sub-Elite Backs	−0.03	−0.07	0.03	0.85	0.12
	Elite Forwards–Sub-Elite Forwards	−0.02	−0.05	0.02	0.85	0.19
**Time on Target** **(Seconds)**	Elite Adjustables–Sub-Elite Adjustables	0.08	−0.01	0.17	0.96	0.05
	Elite Backs–Sub-Elite Backs	−0.02	−0.10	0.05	0.73	0.19
	Elite Forwards–Sub-Elite Forwards	0.02	−0.03	0.08	0.78	0.24
**Response Delay** **(Milliseconds)**	Elite Adjustables–Sub-Elite Adjustables	8.85	−12.51	30.05	0.80	0.13
	Elite Backs–Sub-Elite Backs	12.33	−4.83	28.37	0.92	0.08
	Elite Forwards–Sub-Elite Forwards	12.31	0.13	25.58	0.98	0.03
**Dilation Velocity** **(Seconds)**	Elite Adjustables–Sub-Elite Adjustables	0.04	−0.09	0.17	0.72	0.15
	Elite Backs–Sub-Elite Backs	−0.15	−0.24	−0.04	1.00	0.00
	Elite Forwards–Sub-Elite Forwards	0.07	−0.01	0.15	0.96	0.05
**Peak Constriction Velocity** **(Seconds)**	Elite Adjustables–Sub-Elite Adjustables	0.28	−0.32	0.98	0.82	0.12
	Elite Backs–Sub-Elite Backs	−0.30	−0.75	0.19	0.88	0.12
	Elite Forwards–Sub-Elite Forwards	0.10	−0.28	0.46	0.70	0.28

Estimate represents the median posterior effect size. Lower 95% HDI and Upper 95% HDI denote the bounds of the 95% Highest Density Interval. PD indicates the Probability of Direction. ROPE reflects the proportion of the posterior distribution within the Region of Practical Equivalence.

## Data Availability

The data supporting the findings of this study are not publicly available due to ethical and privacy restrictions involving identifiable athlete performance data. De-identified data may be made available from the corresponding author upon reasonable request and subject to institutional approval.

## Abbreviations

The following abbreviations are used in this manuscript:

AMEDA	Active Movement Extent Discrimination Assessment
AUC	Area Under the Curve
PD	Probability of Direction
PLR	Pupillary Light Reflex
NRLW	National Rugby League Women’s
VVS	Visual-Vestibular System
ROPE	Region of Practical Equivalence
EMM	Estimated Marginal Means
β	Estimate
